# Phonon‐Suppressing Intermolecular Adhesives: Catechol‐Based Broadband Organic THz Generators

**DOI:** 10.1002/advs.202201391

**Published:** 2022-07-15

**Authors:** Ga‐Eun Yoon, Jin‐Hong Seok, Uros Puc, Bong‐Rim Shin, Woojin Yoon, Hoseop Yun, Dongwook Kim, In Cheol Yu, Fabian Rotermund, Mojca Jazbinsek, O‐Pil Kwon

**Affiliations:** ^1^ Department of Molecular Science and Technology Ajou University Suwon 16499 Korea; ^2^ Institute of Computational Physics Zurich University of Applied Sciences (ZHAW) Winterthur 8401 Switzerland; ^3^ Department of Chemistry & Department of Energy Systems Research Ajou University Suwon 443–749 Korea; ^4^ Department of Chemistry Kyonggi University San 94–6, Iui‐dong, Yeongtong‐gu Suwonsi Gyeonggi 443–760 Korea; ^5^ Department of Physics Korea Advanced Institute of Science and Technology (KAIST) Daejeon 34141 Korea

**Keywords:** catechol, nonlinear optics, organic crystals, terahertz waves

## Abstract

Solid‐state molecular phonons play a crucial role in the performance of diverse photonic and optoelectronic devices. In this work, new organic terahertz (THz) generators based on a catechol group that acts as a phonon suppressing intermolecular adhesive are developed. The catechol group is widely used in mussel‐inspired mechanical adhesive chemistry. Newly designed organic electro‐optic crystals consist of catechol‐based nonlinear optical 4‐(3,4‐dihydroxystyryl)‐1‐methylpyridinium (DHP) cations and 4‐(trifluoromethyl)benzenesulfonate anions (TFS), which both have multiple interionic interaction capability. Interestingly, compared to benchmark organic crystals for THz generators, DHP‐TFS crystals concomitantly achieve top level values of the lowest void volume and the highest crystal density, resulting in an exceptionally small amplitude of solid‐state molecular phonons. Simultaneously achieving small molecular phonon amplitude, large optical nonlinearity and good phase matching at infrared optical pump wavelengths, DHP‐TFS crystals are capable of generating broadband THz waves of up to 16 THz with high optical‐to‐THz conversion efficiency; one order of magnitude higher than commercial inorganic THz generators.

## Graphical abstract

New ultra‐broadband organic crystalline THz generators based on catechol intermolecular adhesives have been developed. These crystals present the highest density and the lowest void volume among the present organic THz generators, resulting in exceptionally small amplitude of solid‐state phonon vibrations and high THz conversion efficiency.

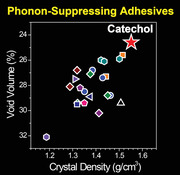

## 1 Introduction

In solid‐state physics, a phonon refers to collective lattice vibrations in solids, in which the constituting particles (atoms, ions) periodically oscillate due to interatomic interactions. Phonon vibrations in principle cannot be avoided, except at absolute zero temperature. In inorganic materials, phonon vibrations strongly influence various physical properties such as optical characteristics at far‐infrared and terahertz (THz) frequencies, thermal and electrical conductivities, and phonon‐coupling phenomena.^[^ ^1^, ^2^, ^3^, ^4^, ^5^, ^6^, ^7^, ^8^, ^9^, ^10^ ^]^ This strong influence of phonon vibrations is also valid for organic materials. For instance, in organic *π*‐conjugated materials, the so‐called molecular phonons play an important role for their functional properties, such as the charge mobility,^[^ ^11^, ^12^, ^13^ ^]^ emission characteristics,^[^ ^14^, ^15^ ^]^ as well as the absorption and refractive index characteristics at far‐infrared and THz frequencies.^[^ ^16^, ^17^, ^18^, ^19^, ^20^, ^21^, ^22^, ^23^, ^24^, ^25^, ^26^, ^27^ ^]^

Compared to inorganic materials, phonon vibrations in organic materials show more complex and strongly anisotropic behavior. While many inorganic materials consist of isotropic sphere‐shaped atoms (and components), most of the organic *π*‐conjugated materials consist of anisotropic non‐sphere‐shape molecules with a rigid *π*‐conjugated bridge having a relatively large size. The amplitude of intermolecular interactions largely differs in different directions and the phonon vibrations in organic materials result from not only whole molecules, but also parts of the molecules.^[^ ^17^, ^28^ ^]^ Different parts of the constituting molecules often vibrate rather independently. It is therefore of high interest to develop a material design tool to control molecular phonon vibrations in organic *π*‐conjugated materials that is specifically targeted to improve the performance of these materials for certain applications.

In THz wave photonics, the influence of phonon vibrations is very large because the optical phonon vibrations occur predominately in the THz frequency range of 0.1–20 THz. In both active THz devices (e.g., THz generators and THz detectors) and passive THz devices (e.g., waveguides, polarizers, and filters), strong phonon vibrations of THz device materials result in the strong absorption of THz waves.^[^ ^29^ ^]^ This absorption mostly limits both the efficiency and the spectral bandwidth of these devices. Therefore, developing new material design approaches for suppressing molecular phonons in organic THz devices is of crucial importance.

**Scheme 1:**
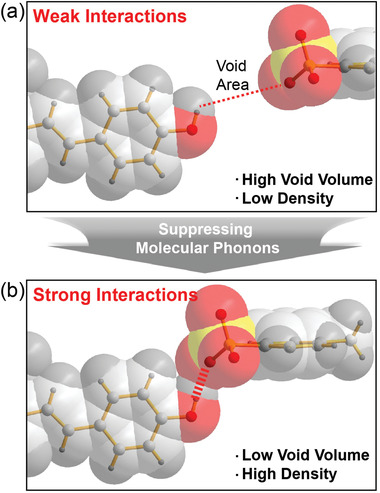
Schematic of self‐assembly with a) weak and b) strong intermolecular (interionic) interactions presented with a) thin and b) thick red dotted lines, respectively. In crystals, strong intermolecular interactions mostly lead to low void volume and high density that may be beneficial for suppressing molecular phonon vibrations.

The aim of this work is to suppress molecular phonon vibrations in organic THz device materials using the approach summarized in **Scheme** **1**. Compared to weak intermolecular interactions in organic THz materials, strong intermolecular interactions may result in lower void volume and higher density. Low void volume is expected to suppress the magnitude of molecular phonon vibrations. Simultaneously, several other properties of organic THz device materials are required for efficient THz generation, as described in more detail in the following section. In Scheme 1, we show an example of intermolecular interactions based on the interionic hydrogen bonds between the phenolic —OH (*δ*+) group and the negatively charged —SO_3_^−^ group. Such hydrogen bonds present main interionic interactions in several promising recently developed organic salt crystals for THz wave generation.^[^ ^16^, ^17^ ^]^ Note that in many organic THz generators, aromatic (i.e., phenolic) and aliphatic —OH groups are incorporated. In this work, we introduce two phenolic —OH groups for the design of new organic THz device materials.

**Figure 1:**
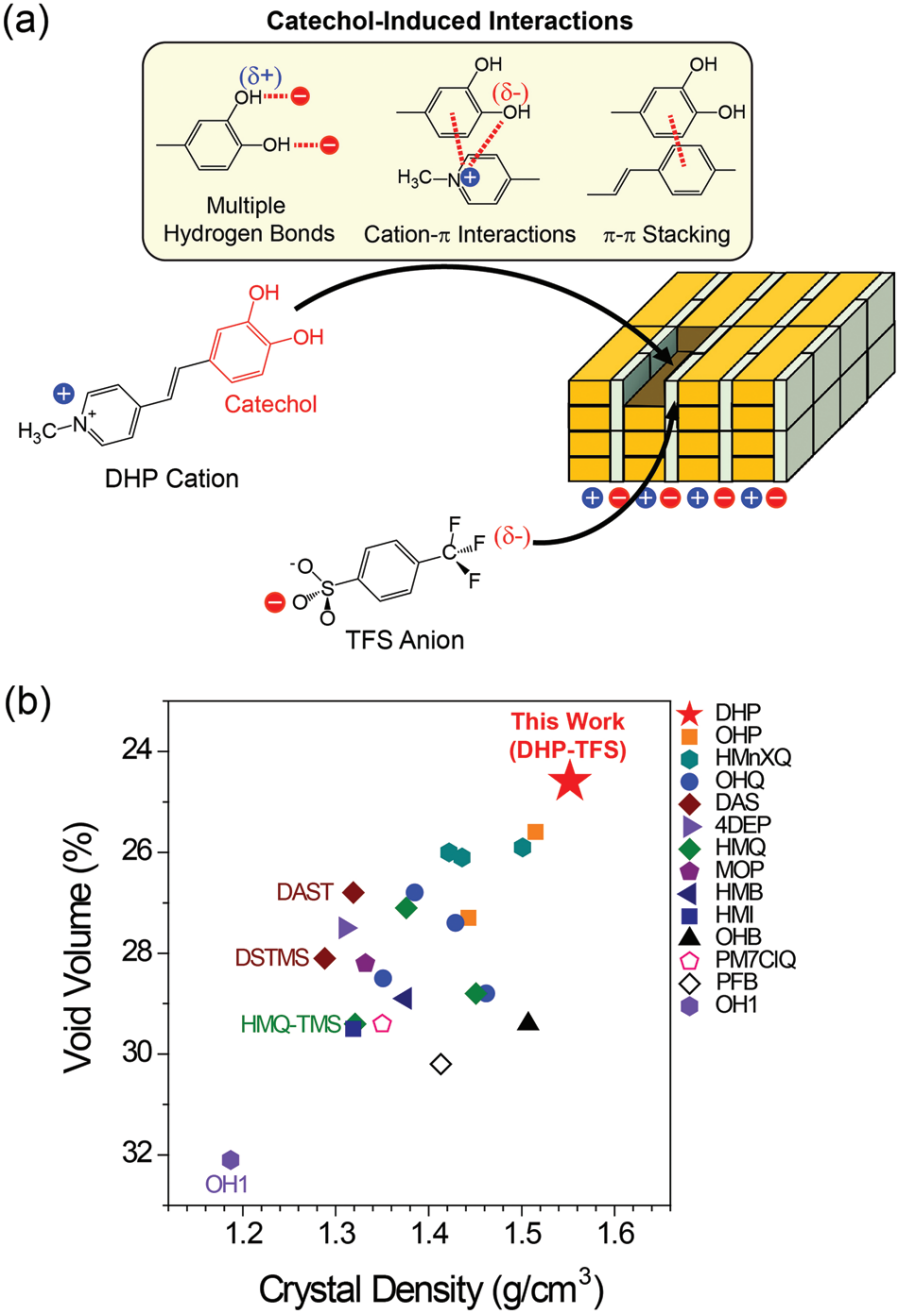
a) Self‐assembly in catechol‐based DHP‐TFS crystals. b) Void volume and crystal density of benchmark organic THz generators (for additional details see Figure S4, Supporting Information).^[^ ^18^, ^19^, ^25^, ^36^, ^38^, ^39^, ^40^, ^41^, ^44^, ^47^, ^50^, ^51^, ^52^, ^53^, ^54^, ^55^, ^56^, ^57^, ^58^, ^59^, ^60^, ^61^, ^62^, ^63^ ^]^

The newly designed organic broadband THz generators are based on catechol possessing two phenolic —OH groups that acts as a phonon suppressing intermolecular adhesive (**Figure** **1**a). The catechol group is widely used in mussel‐inspired mechanical adhesive chemistry due to its multiple intermolecular (interionic) interaction capability.^[^ ^30^, ^31^, ^32^, ^33^, ^34^ ^]^ New 4‐(3,4‐dihydroxystyryl)‐1‐methylpyridinium 4‐(trifluoromethyl)benzenesulfonate (DHP‐TFS) crystals consist of catechol‐containing nonlinear optical cationic chromophore and highly electronegative aromatic matchmaker anions. Compared to benchmark organic nonlinear optical crystals used as efficient THz generators, DHP‐TFS crystals concomitantly exhibit the lowest void volume and the highest crystal density, resulting in exceptionally small absorption coefficient in the THz frequency range. In THz‐wave generation experiments, DHP‐TFS crystals simultaneously achieve a very broad spectral bandwidth (up to 16 THz) and a high conversion efficiency.

## 2 Results and Discussion

### 2.1 Design of Catechol‐Based THz Materials

State‐of‐the‐art organic THz generators are based on organic electro‐optic crystals with a high second‐order optical nonlinearity, exploiting an optical‐to‐THz frequency nonlinear optical conversion process, for example, optical rectification or difference frequency generation.^[^ ^16^, ^35^ ^]^ To simultaneously achieve both broad bandwidth and a high conversion efficiency in organic THz generators, the material design should consider, in addition to other requirements, suppressing the molecular phonon vibrations to reduce the absorption in the THz frequency range. However, this is very difficult because at the same time both a large macroscopic nonlinear optical coefficient and a good phase matching between the optical pump and the generated THz waves must be achieved.

Recently, it has been shown that in organic THz device materials, the amplitude of molecular phonon vibrations is inversely related to void volume and intermolecular interaction strength in crystals as illustrated in Scheme 1.^[^ ^16^, ^17^, ^36^, ^37^, ^38^, ^39^, ^40^ ^]^ For example, as the void volume decreases and the material density increases, the phonon amplitude decreases.^[^ ^39^ ^]^ In organic solids, suppressing molecular phonon vibrations is rather more difficult than enhancing them. Loose packing of molecules gives more space for vibrations than tight packing. Introducing flexible and high steric hindrance groups may readily result in increasing the void volume. In contrast, to decrease the void volume with the tight packing of the constituent molecules in solids, the space‐filling characteristics of molecules and their intermolecular interactions should be simultaneously considered. A higher number density of nonlinear optical chromophores, that result from the tight packing of the molecules, is in addition beneficial for achieving a large macroscopic optical nonlinearity.

To achieve broadband organic THz generators with a high conversion efficiency, DHP‐TFS crystals are newly designed with the catechol‐based nonlinear optical 4‐(3,4‐dihydroxystyryl)‐1‐methylpyridinium (DHP) cation and the highly electronegative aromatic 4‐(trifluoromethyl)benzenesulfonate (TFS) anion (Figure 1a). Introducing multiple intermolecular interaction capable groups in both cations and anions may efficiently suppress molecular phonon vibrations. The catechol group possesses multiple interionic (cation–cation and cation‐anion) interaction capability such as multiple hydrogen bonds, cation–*π* interactions, and face‐to‐face and edge‐to‐face *π*–*π* interactions.^[^ ^30^, ^31^, ^32^, ^33^, ^34^ ^]^ Therefore, catechol group on DHP cation can act as a phonon suppressing intermolecular adhesive. On the other hand, highly electronegative groups (—SO_3_^−^ and —CF_3_) on the TFS anion can also act as multiple interionic interaction capable groups; three negatively charged O atoms on the —SO_3_^−^ group and three partially negatively charged F atoms on the —CF_3_ group.^[^ ^36^, ^37^, ^38^, ^39^ ^]^

In addition, the catechol group acts as an efficient electron donating group. The electron donating strength of the catechol group is large enough for efficient THz generators. Many benchmark organic THz crystals are based on the chromophores with a similar molecular optical nonlinearity of ≈100 × 10^−30^ esu.^[^ ^16^, ^19^, ^39^, ^41^ ^]^ The maximum first hyperpolarizability *β*_max_ of the DHP cationic chromophore is in this work obtained by quantum chemical calculation with the density functional theory (DFT) at the B3LYP/6‐311+G(d,p) level.^[^ ^42^, ^43^ ^]^ The average value of *β*_max_ in the optimized DHP conformers (see Figure S1, Supporting Information) is 121 × 10^−30^ esu. Consequently, the microscopic optical nonlinearity of the DHP cationic chromophore is large enough and comparable to that in other benchmark organic THz crystals.

Besides a large molecular optical nonlinearity of the constituting chromophores, non‐centrosymmetric alignment should be obtained in the crystalline state for obtaining a large macroscopic second‐order nonlinear optical coefficient required for applications. In previous reports, introducing one or two phenolic groups on (cationic) chromophores has shown a strong tendency for a high order parameter with a non‐centrosymmetric alignment of chromophores in the crystalline state, in many cases maximizing the macroscopic optical nonlinearity by a parallel or close‐to parallel alignment of dipolar chromophores.^[^ ^16^, ^19^, ^38^, ^39^, ^40^, ^41^, ^44^, ^45^, ^46^, ^47^ ^]^ The catechol group possesses two phenolic groups. Therefore, catechol‐contained DHP‐TFS crystals may satisfy both a small absorption in the THz frequency range and a large macroscopic nonlinear optical coefficient applicable for broadband efficient organic THz generators.

### 2.2 Exceptionally Small Molecular Phonon Amplitudes

**Figure 2:**
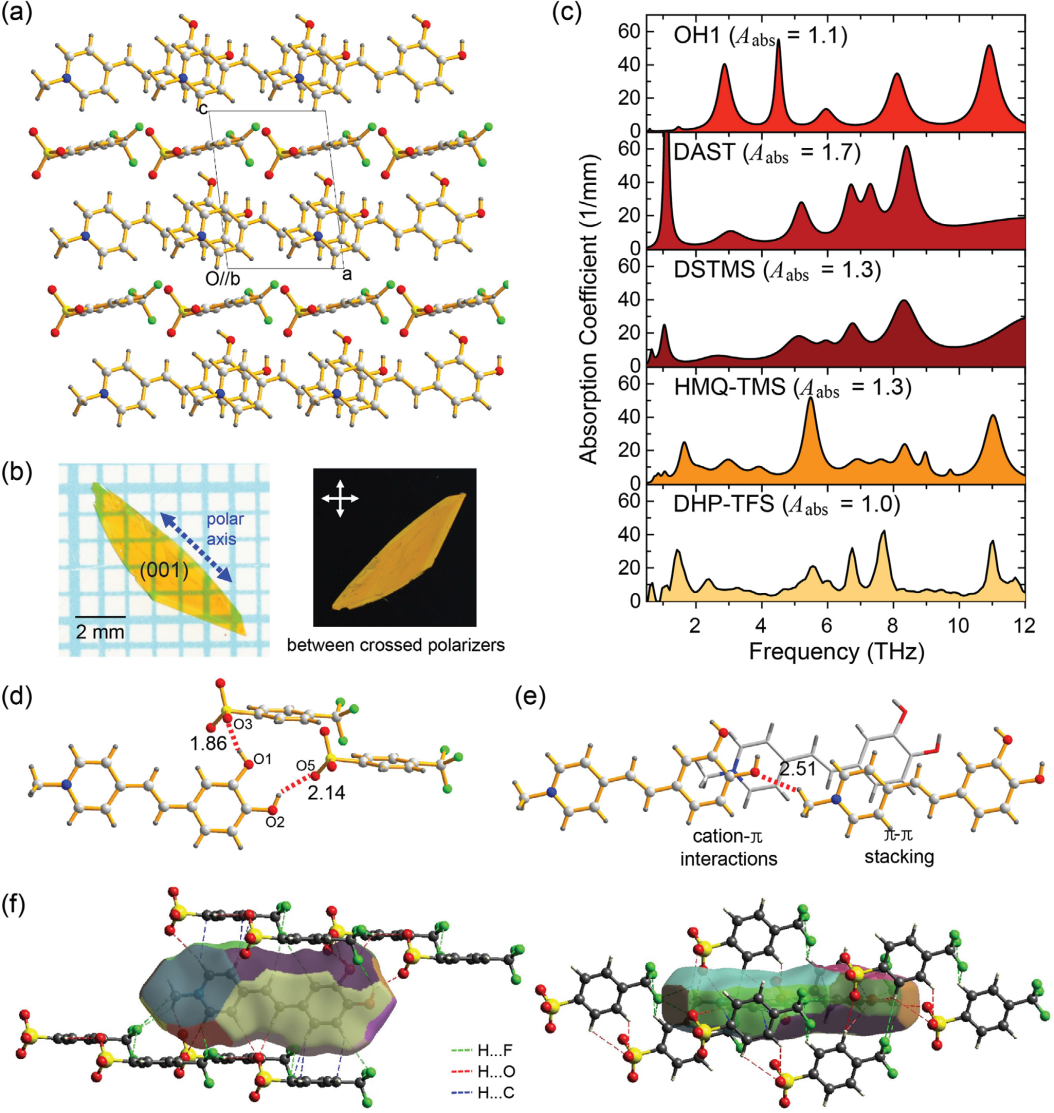
a) Molecular alignment of DHP‐TFS crystals. b) Photographs of an as‐grown DHP‐TFS crystal in transmission mode (left: on a transparent grid sheet and right: between crossed polarizers). c) Absorption coefficient along the polar axis of benchmark organic THz generator and DHP‐TFS.^[^ ^20^, ^26^, ^64^, ^65^, ^66^, ^67^, ^68^, ^69^ ^]^ The relative absorption ratio (*A*_abs_) is here defined as the ratio of the integrated absorption coefficient relative to DHP‐TFS crystals in the range of 1–12 THz. d) Catechol‐induced strongest hydrogen bonds between DHP cations and TFS anions and e) interactions between DHP cations. f) Hirshfeld surface (fragment patch) of DHP cation with the atom contacts of H⋅⋅⋅F, H⋅⋅⋅O, and H⋅⋅⋅C (<3.0 Å).

In a qualitative powder second harmonic generation (SHG) measurement,^[^ ^48^, ^49^ ^]^ DHP‐TFS shows a strong SHG signal (Figure S2, Supporting Information). This shows that DHP cationic chromophores are aligned in a non‐centrosymmetric way in the crystalline state, which was also confirmed by X‐ray single crystal structure analysis. **Figure** **2**a shows the molecular alignment in DHP‐TFS crystals. DHP‐TFS crystals possess non‐centrosymmetric space group *P*1 with a parallel‐type cation–anion assembly;^[^ ^16^ ^]^ DHP cation layers are altered with TFS anion layers.

Interestingly, DHP‐TFS crystals exhibit a very low void volume (24.6% of the total volume, see Figure S3, Supporting Information) and a high crystal density (1.552 g cm^−3^). Figure 1b shows the void volume and the crystal density of DHP‐TFS crystals and benchmark organic THz generators (see also Figure S4, Supporting Information, for additional details).^[^ ^18^, ^19^, ^25^, ^36^, ^38^, ^39^, ^40^, ^41^, ^44^, ^47^, ^50^, ^51^, ^52^, ^53^, ^54^, ^55^, ^56^, ^57^, ^58^, ^59^, ^60^, ^61^, ^62^, ^63^ ^]^ Among many organic nonlinear optical crystals reported, in Figure 1b we select organic crystals with: i) showing a large (state‐of‐the‐art) optical nonlinearity (e.g., two aromatic ring‐based stilbene and polyene chromophores); ii) reported crystal structure at near room temperature; iii) reported THz wave generation characteristics; and iv) introducing only H, F and Cl substituents on aromatic rings. As shown in Figure 1b, DHP‐TFS crystals exhibit both the lowest void volume and the highest crystal density. This may lead to a strong suppression of molecular phonon vibrations and result in a small amplitude of solid‐state molecular phonons (i.e., low absorption in the THz frequency range).

**Figure 3:**
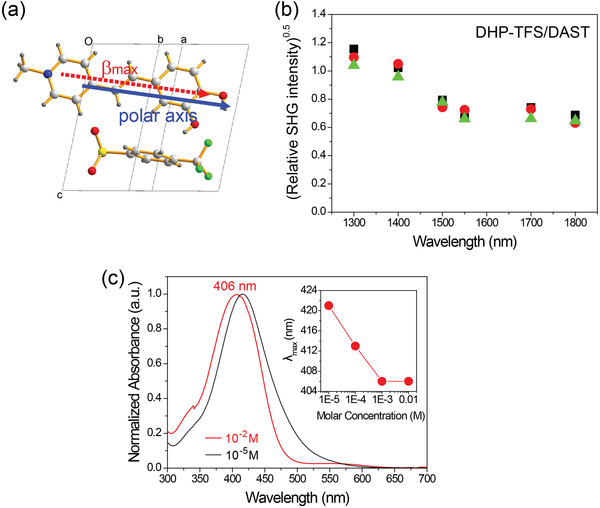
a) Ordering of the DHP chromophore in DHP‐TFS crystals; view along the direction normal to the direction of *β*_max_ and normal to the crystallographic axis *c*. The direction of *β*_max_ as determined by DFT makes an angle of ≈7° to the (001) plane (*ab* plane) of an as‐grown DHP‐TFS crystal. b) The square root of the relative SHG intensity of DHP‐TFS powders relative to that of DAST powders as a function of the fundamental pump wavelength. Three symbols (triangle, circle, and square) present the measurements with differently grinded DHP‐TFS samples. c) Normalized absorption spectra of DHP‐TFS in methanol at different molar concentrations (inset: the corresponding wavelength of maximum absorption *λ*_max_ as a function of molar concentration).

Bulk single crystals of DHP‐TFS were successfully grown by a slow cooling method in methanol as shown in Figure 2b. As‐grown DHP‐TFS crystals show good optical quality with high homogeneity and large enough for optical experiments. The crystals were grown in a plate‐like morphology with the largest crystal facet along the *ab*‐crystallographic plane, that is, the (001) plane, as shown in Figure 2b. Since the direction of *β*_max_ and the polar axis as determined by DFT calculations is practically along this plane (aligned at only ≈7° with respect to this plane, see **Figure** **3**a), the as‐grown morphology is close‐to optimal to access the largest component of the nonlinear optical susceptibility. The following linear optical measurements (the absorption coefficient in the THz frequency range) and the nonlinear optical measurements (THz wave generation) were performed with as‐grown DHP‐TFS crystals without additional processing. In all experiments reported in this work, the optical and the THz wave polarization are parallel to the projection of the polar axis to the (001) crystal plane (the dotted blue arrow in Figure 2b).

Figure 2c shows the measured absorption coefficient of DHP‐TFS crystals in the THz frequency range up to 12 THz. For comparison, Lorentz absorption curves of previously measured benchmark organic THz generators are also included.^[^ ^20^, ^26^, ^64^, ^65^, ^66^, ^67^, ^68^, ^69^ ^]^ In Figure 2c, 2‐(3‐(4‐hydroxystyryl)‐5,5‐dimethylcyclohex‐2‐enylidene)malononitrile (OH1) crystal is selected because it represents the non‐ionic organic THz generators having phenolic groups.^[^ ^47^ ^]^ 4‐(4‐(dimethylamino)styryl)‐1‐methylpyridinium 4‐methylbenzenesulfonate (DAST) and 4‐(4‐(dimethylamino)styryl)‐1‐methylpyridinium 2,4,6‐trimethylbenzenesulfonate (DSTMS) crystals represent ionic organic THz generators without phenolic groups, while 2‐(4‐hydroxy‐3‐methoxystyryl)‐1‐methylquinolinium 2,4,6‐trimethylbenzenesulfonate (HMQ‐TMS) crystal represents ionic organic THz generators having phenolic groups.^[^ ^16^, ^25^, ^26^, ^50^, ^51^, ^52^ ^]^ All included ionic benchmark crystals (DAST, DSTMS, and HMQ‐TMS) exhibit a parallel‐type cation–anion assembly,^[^ ^16^ ^]^ similar with the newly developed DHP‐TFS crystals.

As shown in Figure 2c, in the broad THz frequency range measured, DHP‐TFS crystals exhibit exceptionally small overall absorption with weaker absorption peaks compared to benchmark organic THz crystals. This indicates a smaller amplitude of the solid‐state molecular phonons in DHP‐TFS crystals. The relative absorption ratio (*A*_abs_), which is here defined as the ratio of the integrated absorption coefficient relative to DHP‐TFS crystals in the range of 1–12 THz is 1.0 for DHP‐TFS, 1.1 for OH1, 1.7 for DAST, 1.3 for DSTMS and 1.3 for HMQ‐TMS, where the relative error of these values is ≈10%. The strongest absorption peaks are relatively smaller in DHP‐TFS crystals compared to OH1 and DAST crystals. Note that the inorganic THz generator ZnTe exhibits a very strong absorption peak (>1000 mm^−1^) due to the transverse optical phonon mode at 5.3 THz.^[^ ^9^ ^]^

In previous studies, molecular vibrations of ionic nonlinear optical crystals (HMQ‐TMS) showing parallel‐type cation–anion assembly were classified into three frequency regions: the R1 region mainly includes entire molecular motions (translations and rotations); the R2 region includes both molecular motions and intramolecular vibrations; and the R3 region includes intramolecular vibrations.^[^ ^17^ ^]^ For HMQ‐TMS crystals, R1, R2 and R3 regions were defined at below ≈2, ≈2–5 and above ≈5 THz, respectively. The boundary frequencies between these three regions of molecular vibrations may vary for different organic THz generators. Since DHP‐TFS crystals exhibit a parallel‐type cation–anion assembly like HMQ‐TMS (also DAST and DSTMS), this classification may still be valid. We here refer to these three regions (R1, R2, and R3) as low, medium, and high THz frequency regions, respectively.

Compared to DAST crystals, DHP‐TFS crystals exhibit a lower overall absorption in all low, medium, and high THz frequency regions. Compared to DSTMS and HMQ‐TMS crystals, the overall absorption of DHP‐TFS crystals is lower at least in medium and high THz frequency regions (R2 and R3 regions; 2–12 THz). In comparison to analogous 4‐(4‐hydroxystyryl)‐1‐methylpyridinium‐1‐ium 4‐(trifluoromethyl)benzenesulfonate (OHP‐TFS) crystals with also a high density and a low void volume (see Figure S4, Supporting Information),^[^ ^39^ ^]^ DHP‐TFS crystals exhibit a lower overall absorption in the medium THz frequency region (R2 region; 2–6 THz) as shown in Figure S7, Supporting Information. On the other hand, in the high THz frequency region; 6–12 THz, DHP‐TFS crystals exhibit a similar absorption with OHP‐TFS crystals.^[^ ^39^ ^]^

This exceptionally small amplitude of solid‐state molecular phonons in DHP‐TFS crystals (relatively low absorption peaks in the THz frequency range) is related to top level values of both the lowest void volume and the highest crystal density (Figure 1b). In addition, the catechol group acting as a phonon suppressing intermolecular adhesive on DHP cations may also strongly contribute to the lower absorption. As shown in Figure 2d, the catechol group on the DHP cation forms two strong hydrogen bonds (between the —OH⋅⋅⋅^−^O_3_S— groups) with two TFS anions, which reduces the void volume (Figure S3, Supporting Information). In DHP cation layers, the catechol group contributes to many strong secondary bonds; the hydrogen bond between the HO⋅⋅⋅H_3_C—N— groups with a distance of ≈2.5 Å, cation–*π* interactions, face‐to‐face *π*–*π* stacking interactions (Figure 2e). As shown in Figure 2f with the Hirshfeld surface^[^ ^70^, ^71^, ^72^ ^]^ of the DHP cation, cation–anion and anion–anion assemblies consist of many strong secondary bonds between H⋅⋅⋅F, H⋅⋅⋅O and H⋅⋅⋅C (<3.0 Å). The corresponding 2D fingerprint plots of the Hirshfeld surface analysis for the DHP cation and the TFS anion are presented in Figures S5 and S6, Supporting Information, respectively. Therefore, introducing multiple intermolecular interaction capable groups (e.g., the catechol group) is an efficient tool to suppress molecular vibrations in organic crystals.

### 2.3 Efficient Broadband THz Wave Generators

To simultaneously achieve a broad bandwidth and a high conversion efficiency with organic THz generators, beside a small amplitude of molecular phonon vibrations (i.e., low absorption peaks in the THz range) discussed above, organic crystals should concomitantly exhibit a large macroscopic nonlinear optical coefficient and a good phase matching between the generated THz waves and the optical pump wavelength used. In DHP‐TFS crystals, nonlinear optical DHP chromophores show a perfectly parallel alignment with the polar axis (Figure 3a), which is due to the triclinic *P*1 crystal symmetry with only one molecular pair (cation and anion) in the unit cell. This parallel chromophore ordering implies that DHP‐TFS crystals have a maximized diagonal macroscopic nonlinear optical coefficient without any loss of molecular nonlinearity. The maximum first hyperpolarizability *β*_max_ of DHP cationic chromophore, calculated with DFT for the molecular conformation as determined in the X‐ray structural analysis—the so‐called experimental conformer (EXP)—is 132 × 10^−30^ esu. This value is slightly different to that of the two conformers (OPT1 and OPT2, see Figure S1, Supporting Information) optimized by DFT (118 and 124 × 10^−30^ esu) because of the slightly different molecular conformation in the crystal affected by intermolecular interactions. Consequently, the diagonal component of the effective first hyperpolarizability tensor $\beta_{111}^{\mathrm{eff}}$ of DHP‐TFS crystals is ≈132 × 10^−30^ esu. This value is similar to the values of the largest component of the effective hyperpolarizability tensor in several benchmark organic‐crystal THz generators.^[^ ^16^, ^19^, ^39^, ^41^ ^]^

To confirm the high macroscopic optical nonlinearity experimentally, the SHG conversion efficiency of DHP‐TFS powders is compared to the well‐known benchmark organic DAST crystals. Figure 3b shows the square root of the relative SHG intensity of DHP‐TFS powders relative to that of DAST powders ((*I*_DHP‐TFS_/*I*_DAST_)^0.5^) as a function of the fundamental pump wavelength. Note that the effective first hyperpolarizability is roughly proportional to the square root of the SHG intensity. In the non‐resonant regime far away from electronic resonances (i.e., for fundamental pump wavelengths ≥ 1500 nm), the average value of (*I*_DHP‐TFS_/*I*_DAST_)^0.5^ is ≈0.7. This value is very similar to the relative ratio of the diagonal component of the effective first hyperpolarizability tensor $\beta_{111}^{\mathrm{eff}}$ (≈0.8); 132 × 10^−30^ esu for DHP‐TFS and 161 × 10^−30^ esu for DAST. Consequently, the value for the diagonal effective first hyperpolarizability tensor element $\beta_{111}^{\mathrm{eff}}$ of DHP‐TFS crystals (132 × 10^−30^ esu) obtained from DFT calculations correlates very well to the results of the powder SHG measurements.

Theoretical DFT calculations and experimental SHG measurements (Figure 2b) show that the second‐order optical nonlinearity of DHP‐TFS crystals is roughly of the same order of magnitude as that of DAST crystals (e.g., the second‐order nonlinear optical coefficient *χ* ^(2)^ = 2020 and 580 pm V^−1^ at 1318 and 1542 nm, respectively and the electro‐optic coefficient *r*_11_ = 77, 53 and 47 pm V^−1^ at 800, 1313 and 1535 nm, respectively^[^ ^73^, ^74^ ^]^). This large second‐order optical nonlinearity of DHP‐TFS crystals compares favorably to other‐types of nonlinear optical and THz generation materials.^[^ ^16^, ^65^, ^75^, ^76^, ^77^ ^]^ For example, the electro‐optic coefficient of widely used inorganic THz crystals (e.g., ZnTe, GaAs, and GaP) is one order of magnitude smaller than that of the organic benchmarks.^[^ ^65^, ^77^ ^]^ Note also that compared to the recently proposed halide perovskite crystals for nonlinear optics, DHP‐TFS crystals exhibit much higher second‐order optical nonlinearity.^[^ ^75^, ^76^ ^]^

Organic crystals with a yellow color often exhibit good phase matching characteristics for THz wave generation when using both near‐infrared and infrared optical pump wavelengths.^[^ ^41^, ^55^ ^]^ The chromophores used in yellow‐color organic THz generators exhibit the wavelength of maximum absorption *λ*_max_ in the range of 390–420 nm in methanol. Figure 3c shows the absorption spectra of DHP‐TFS in methanol and the corresponding wavelength of maximum absorption *λ*_max_ in the inset. To avoid the contribution of the phenolate form that only exists in solution,^[^ ^55^ ^]^ various concentrations were examined. The wavelength of maximum absorption *λ*_max_ of DHP‐TFS is ≈406 nm in methanol, which is in the yellow‐color regime.

**Figure 4:**
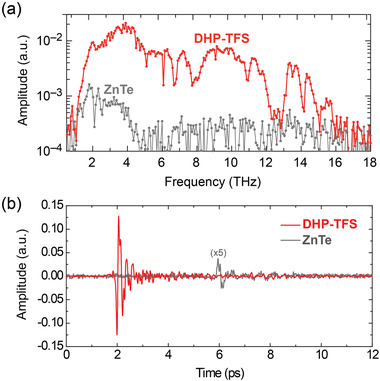
THz wave generation in a 0.15 mm thick (001) DHP‐TFS and a 1.0 mm thick (110) ZnTe at 1560 nm pump wavelength and 38 fs pulse width: a) frequency domain measured with the resolution of 0.085 THz and b) the corresponding time‐domain signals. Note that for ZnTe in Figure 4b, the amplitude was multiplied by a factor of 5 compared to DHP‐TFS for clarity (the measured peak‐to‐peak signal amplitude is ≈0.0125 V for ZnTe and ≈0.25 V for DHP‐TFS).

**Figure** **4** shows the results of THz wave generation in DHP‐TFS based on the nonlinear optical process of optical rectification. We employed a 0.15 (1 ± 10%) mm thick (001) DHP‐TFS using 1560 nm pump pulses with the pulse width of 38 fs (see also Supporting Information for the experimental details). The THz generation characteristics are compared to those of a 1.0 mm thick inorganic (110) ZnTe crystal, which is one of the widely used inorganic standard THz generators based on optical rectification in the simplest collinear geometry.^[^ ^16^ ^]^ All THz measurements were performed at room temperature in dry air with the relative humidity of ≈3%. ZnTe is the standard inorganic electro‐optic crystal for THz wave generation, however it has a relatively low nonlinear optical coefficient compared to organics and its phase matching characteristics are in addition not optimal at 1560 nm. Note that this is a very interesting wavelength because of the availability of compact and relatively low‐cost fiber femtosecond lasers at 1560 nm.

As shown in Figure 4a, DHP‐TFS crystals on the other hand satisfy the phase matching very well in the very broad THz range; the generated THz waves include frequencies up to ≈16 THz. This THz bandwidth of DHP‐TFS crystals is over 4 times higher than that of ZnTe crystals. The optical‐to‐THz conversion efficiency is also extremely large; in time domain (Figure 4b), the peak‐to‐peak THz amplitude generated in DHP‐TFS is ≈20 times higher than the amplitude generated in ZnTe. Both measurements were done using the same delay range of the probe fs pulse compared to the pump beam; the shift of the signal for ZnTe by ≈4 ps is due to the different thickness and the different refractive index of both THz generators. These first results of THz‐wave generation with DHP‐TFS are very promising considering that the present crystals are still very thin and the electric field at phase matching is expected to rise proportionally with the thickness.^[^ ^16^ ^]^

**Figure 5:**
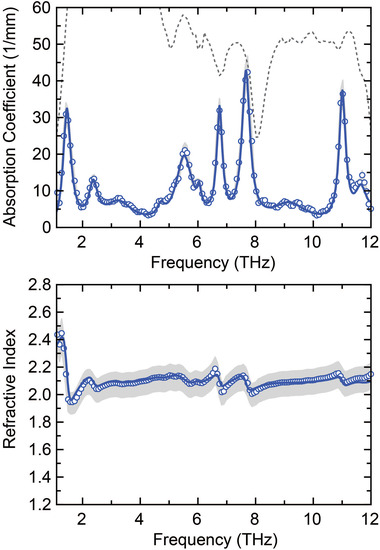
Correlation between the optical properties (absorption coefficient and refractive index) along the polar axis of DHP‐TFS crystals in the THz frequency range. The optical properties (open circles) were measured for a 0.15 (1 ± 10%) mm thick DHP‐TFS crystal. The grey shaded area in both figures presents a possible range of values due to the 10% uncertainty in the thickness, for which the refractive index is much more sensitive than the absorption coefficient. The dotted grey curve shows the dynamic range of the measurement, that is, the maximum measurable absorption for a 0.15 mm thick crystal. The solid curve is according to the Lorentz multiple oscillator model (see Supporting Information).

The spectral shape and the amplitude of the generated THz waves of DHP‐TFS crystals are strongly correlated with the absorption and the refractive index characteristics in the THz frequency range. The refractive index varies following the absorption peaks in the THz frequency range according to the usual Kramers–Kronig relations. In general, strong absorption peaks result in strong oscillation of the refractive index profile (dispersion), which is for organic nonlinear optical crystals usually stronger in the lower THz frequency range. For providing more information for THz wave generation characteristics, we measured the refractive index and the absorption coefficient along the polar axis of DHP‐TFS crystals as a function of THz frequency, as shown in **Figure** **5**. The smaller the THz absorption peaks the weaker the oscillations of the refractive index. Small variations of the refractive index are important to achieve a good phase matching between the optical pump and the generated THz waves in a broad THz frequency range.

The measured spectra shown in Figure 4a present the Fourier transform of the measured time‐domain signals shown in Figure 4b with the resolution of the measurement (0.085 THz), without any additional processing. The small oscillations observed in the generated spectrum are due to the residual water vapor absorption at the relative humidity of the measurement (≈3%), see also Figure S8, Supporting Information. As shown in Figure S8, Supporting Information, the shape of the measured THz spectrum in DHP‐TFS crystals is inversely matched with the overall shape of the absorption coefficient curve in both the generation and the detection organic crystals. The peak positions in the absorption coefficient spectrum are correlated with the position of dimples in the measured THz spectrum, which are also due to self‐absorption of the generated THz waves by DHP‐TFS crystals. The relatively low absorption of the vibrational modes with the weak refractive index dispersion of DHP‐TFS in the THz frequency range is here directly visible as the reduction of the spectral dimples and therefore a flatter spectrum as a function of frequency, which is beneficial for THz photonics applications.

## 3 Conclusion

In summary, we have designed new organic THz generators based on the catechol group that acts as a phonon‐suppressing intermolecular adhesive. Compared to organic crystals used as benchmark THz generators, DHP‐TFS crystals concomitantly achieve top level values of the lowest void volume and the highest crystal density. This results in exceptionally small absorption in the THz frequency range, which greatly limits the self‐absorption of the THz waves generated by DHP‐TFS crystals. Consequently, DHP‐TFS crystals show excellent THz wave generation performance, characterized by a very high bandwidth (up to 16 THz) and a high efficiency. Therefore, introducing phonon‐suppressing groups such as the catechol group, capable of multiple intermolecular interactions, is an interesting material design tool with a high potential in the fields of fundamental research and applications that strongly depend on the fundamental material phonon characteristics.

## 4 Experimental Section

The details of the synthesis, DFT calculations, SHG measurements, crystal characteristics, THz generation, THz absorption, and THz refractive index measurements are described in the Supporting Information.

## Conflict of Interest

The authors declare no conflict of interest.

## Supporting information

Supporting Information

## Data Availability

Research data are not shared.
