# Supplementary material for: Phonon‐Suppressing Intermolecular Adhesives: Catechol‐Based Broadband Organic THz Generators
Source: Adv Sci (Weinh). 2022 Jul 15;9(24):2201391. doi: 10.1002/advs.202201391 (PMC9403645; doi:10.1002/advs.202201391)
Supplement: Supplementary file 1 — Supporting Information [file ADVS-9-2201391-s001.pdf]

ADVANCED SCIENCE
Open Access

Supporting Information

for *Adv. Sci.*, DOI 10.1002/advs.202201391

Phonon-Suppressing Intermolecular Adhesives: Catechol-Based Broadband Organic THz Generators

Ga-Eun Yoon, Jin-Hong Seok, Uros Puc, Bong-Rim Shin, Woojin Yoon, Hoseop Yun, Dongwook Kim, In Cheol Yu, Fabian Rotermund, Mojca Jazbinsek and O-Pil Kwon**

Copyright WILEY-VCH Verlag GmbH & Co. KGaA, 69469 Weinheim, Germany, 2018.

Supporting Information

Phonon-Suppressing Intermolecular Adhesives: Catechol-Based Broadband Organic THz Generators

Ga-Eun Yoon[†]*, Jin-Hong Seok*[†]*, Uros Puc*[†]*, Bong-Rim Shin, Woojin Yoon, Hoseop Yun, Dongwook Kim, In Cheol Yu, Fabian Rotermund, Mojca Jazbinsek*, O-Pil Kwon**

G. E. Yoon, J. H. Seok, B. R. Shin, Prof. O. P. Kwon
Department of Molecular Science and Technology, Ajou University, Suwon 443-749 (Korea)
E-mail: opilkwon@ajou.ac.kr
Dr. U. Puc, Dr. M. Jazbinsek
Institute of Computational Physics, Zurich University of Applied Sciences (ZHAW), 8401 Winterthur (Switzerland)
E-mail: mojca.jazbinsek@zhaw.ch
W. Yoon, Prof. H. Yun
Department of Chemistry & Department of Energy Systems Research, Ajou University, Suwon 443-749 (Korea)
Prof. D. Kim
Department of Chemistry, Kyonggi University, San 94-6, Iui-dong, Yeongtong-gu, Suwonsi, Gyeonggi 443-760 (Korea)
I. C. Yu, Prof. F. Rotermund
Department of Physics, Korea Advanced Institute of Science and Technology (KAIST), Daejeon 34141 (Korea)

[†]These authors contributed equally to this work.

A. Synthesis

4-(3,4-Dihydroxystyryl)-1-methylpyridinium 4-(trifluoromethyl)benzenesulfonate (DHP-4TFS): 1,4-Dimethylpyridinum 4-(trifluoromethyl)benzenesulfonate (7 g, 21 mmol) and 3,4-dihydroxybenzaldehyde (2.9 g, 21 mmol) were dissolved in ethanol (100 mL) and then piperidine was added (0.42 ml, 4.2 mmol). The solution was stirred at 75 °C for 46 h. After cooling to room temperature, black powder was eliminated by filtration. After evaporating the solvent, yellow powder was obtained by precipitating with 1,2-dimethoxyethane. The crystalline powder was obtained by recrystallization in methanol and dried in vacuum oven at 105 °C overnight. Yield = 63 %. ^{1}H NMR (600 MHz, DMSO-d_6, δ): 8.74 (d, 2H, J = 6.6 Hz, C_5H_4N), 8.11 (d, 2H, J = 6.6 Hz, C_5H_4N), 7.84 (d, 1H, J = 15.8 Hz, CH), 7.79 (d, 2H, J = 7.8 Hz, $C_6H_4SO_3^-$), 7.70 (d, 2H, J = 7.8 Hz, $C_6H_4SO_3^-$), 7.15 (d, 1H, J = 15.6 Hz, CH), 7.14 (d, 1H, J = 1.8 Hz, $C_6H_5O_2$), 7.06 (m, 1H, $C_6H_5O_2$), 6.82 (d, H, J = 8.4 Hz, $C_6H_5O_2$), 4.20 (s, 3H, NCH_3). ^{13}C NMR (600 MHz, DMSO-d_6, δ): 153.02, 152.02, 148.74, 145.79, 144.52, 141.58, 128.91, 126.84, 126.80, 126.36, 125.04, 124.89, 123.23, 122.79, 121.56, 121.43, 119.48, 115.96, 114.75, 46.58. Elemental analysis for $C_{21}H_{18}F_3NO_5S$: Calcd. C 55.63, H 4.00, F 12.57, N 3.09, O 17.64, S 7.07; Found: C 55.52, H 4.09, N 3.18, S 6.88.

B. Microscopic Optical Nonlinearity of DHP Cations

The quantum chemical calculation with density functional theory (DFT) at the B3LYP/6-311+G(d,p) level were performed for calculating the maximum first hyperpolarizability β_{max} of DHP cationic chromophore, similar as in Ref. [*J. Phys. Chem. C* **2008**, *112*, 7846; *CrystEngComm*, **2011**, *13*, 444].

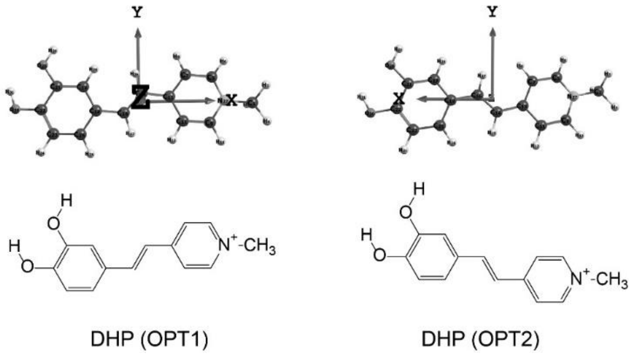

Figure S1. Optimized molecular conformation (OPT) of two isomers of DHP cationic chromophores that have different directions between the dihydroxyl group and the pyridinium ring. The maximum first hyperpolarizability β_{max} of the optimized DHP conformers is 118 and 124 × 10^{-30} esu for DHP (OPT1) and DHP (OPT2), respectively. The average β_{max} of two DHP conformers is 121 × 10^{-30} esu.

C. Powder SHG Test

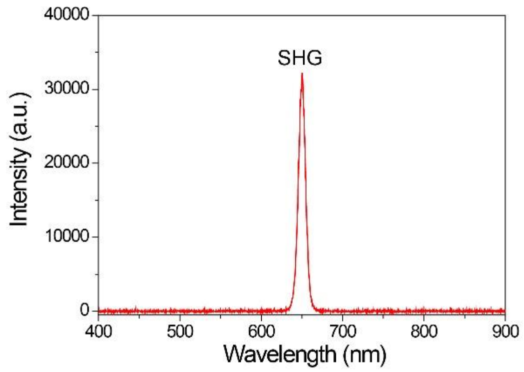

Figure S2. Powder second harmonic generation (SHG) test of DHP-TFS powder recrystallized in methanol. At the fundamental wavelength of 1300 nm, strong SHG signal at 650 nm was observed by using a spectrometer.

D. Crystal Characteristics

Crystal structure of DHP-TFS: DHP-TFS single crystals for X-ray crystal structure analysis were grown by slow evaporation method in methanol at 32 °C. $C_{14}H_{14}NO_2 \cdot C_7H_4F_3O_3S$, M_r = 453.42, triclinic, space group *P*1, *a* = 6.5896(4) Å, *b* = 8.3110(6) Å, *c* = 9.1480(6) Å, α = 101.717(2)°, *β* = 95.487(2)°, γ = 95.338(2)°, *V* = 485.05(6) $Å^3$, *Z* = 1, *T* = 290(1) K, μ(MoKα)= 0.23 mm^{-1}. Of 4756 reflections collected in the θ range 3.0-27.4° using ω scans on a Rigaku R-axis Rapid S diffractometer, 3614 were unique reflections (R_{int} = 0.018). The structure was solved and refined against F^2 using SHELXL-2018/3, [G. M. Sheldrick (2015). Acta Cryst. C71, 3] 283 variables, wR_2 = 0.073, R_1 = 0.027 ($F_o^2 > 2\sigma(F_o^2)$), GOF = 1.05, and max/min residual electron density 0.19/-0.19 $eÅ^{-3}$. CCDC-2073293.

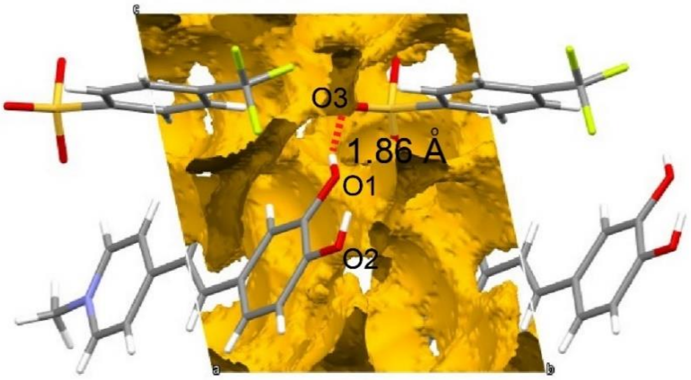

Figure S3. Void shape of DHP-TFS crystals, as viewed along the *a*-axis. [Mercury 4.3.0 program, Cambridge Crystallographic Data Centre (CCDC), https://www.ccdc.cam.ac.uk] The void volume of DHP-TFS crystals is very low, 24.6% of the total volume. Due to strong hydrogen bonds between the catechol group on the DHP cation (-OH) and the TFS anion ($-SO_3^-$) (O1-H···$^-$O3-S- and O2-H···$^-$O5-S- groups) that are more clearly shown in Figure 2d, the area around the catechol group exhibits a relatively very low void volume.

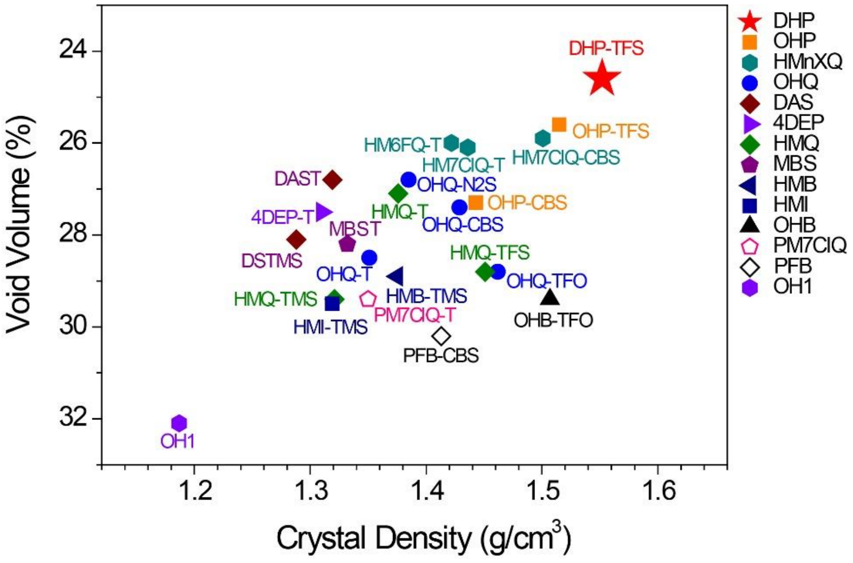

Figure S4. Void volume and crystal density of state-of-the-art organic THz generators: DHP-TFS [this work], OH1 [*Adv. Funct. Mater*., **2008**, *18*, 3242 (CCDC-672263)], DAST [*Science*, **1989**, *245*, 626, *Chem. Mater*., **1994**, *6*, 1137 (CCDC-1175744)], DSTMS [*Adv. Funct. Mater*. **2007**, *17*, 2018(CCDC-277597)], HMQ-TMS [*Sci. Rep.* **2013**, *3*, 3200 (CCDC-931931)], OHP-TFS [*Adv. Opt. Mater*., **2021**, *9*, 2100618 (CCDC-2018182)], PFB-CBS [*Adv. Opt. Mater*. **2020**, *8*, 1901921 (CCDC-1839763)], PM7ClQ-T [*Adv. Opt. Mater*., DOI: 10.1002/adom.202100324 (CCDC-1950430)], HMI-TMS [*Adv. Opt.Mater*. **2020**, *8*, 1902099 (CCDC-1848730)], HMB-TMS [*Adv. Mater*. **2017**, *29*, 1701748 (CCDC-1536610)], OHB-TFO [*Adv. Sci*. **2020**, *7*, 2001738 (CCDC-1885049)], OHQ-T [*Adv. Opt. Mater*. **2015**, *3*, 756 (CCDC-1024236)], HMQ-TFS [*Adv. Funct. Mater*. **2017**, *27*, 1605583 (CCDC-1008220)], OHQ-TFO [*Adv. Opt. Mater*. **2019**, *7*, 1900953 (CCDC-1872614)], 4DEP-T [*Bull. Chem. Soc. Jpn*. **2005**, *78*, 344 (CCDC-235903), *Adv. Opt. Mater*. **2018**, *6*, 1800383], MBST [*J. Phys. Chem. Solids*. **2011**, *72*, 1002 (CCDC-774844); *Opt. Mater*. **2021**, *117*, 111119], HMQ-T [*Adv. Funct. Mater*. **2012**, *22*, 200 (CCDC-824804)] OHQ-CBS [*Adv. Opt. Mater*. **2018**, *6*, 1700930 (CCDC-1441587)], OHQ-N2S [*Adv. Opt. Mater*. **2017**, *5*, 1600758 (CCDC-1008221)], OHP-CBS [*Adv. Funct. Mater*. **2018**, *28*, 1801143 (CCDC-1586163)], HM6FQ-T [*Adv. Opt. Mater*. **2019**, *7*, 1801495 (CCDC-1848136)], HM7ClQ-T [*Adv. Opt. Mater*.

2020, *8*, 1901840 (CCDC-1939794)], HM7ClQ-CBS [*Adv. Opt. Mater*. **2020**, *8*, 1901840 (CCDC-1939795)].

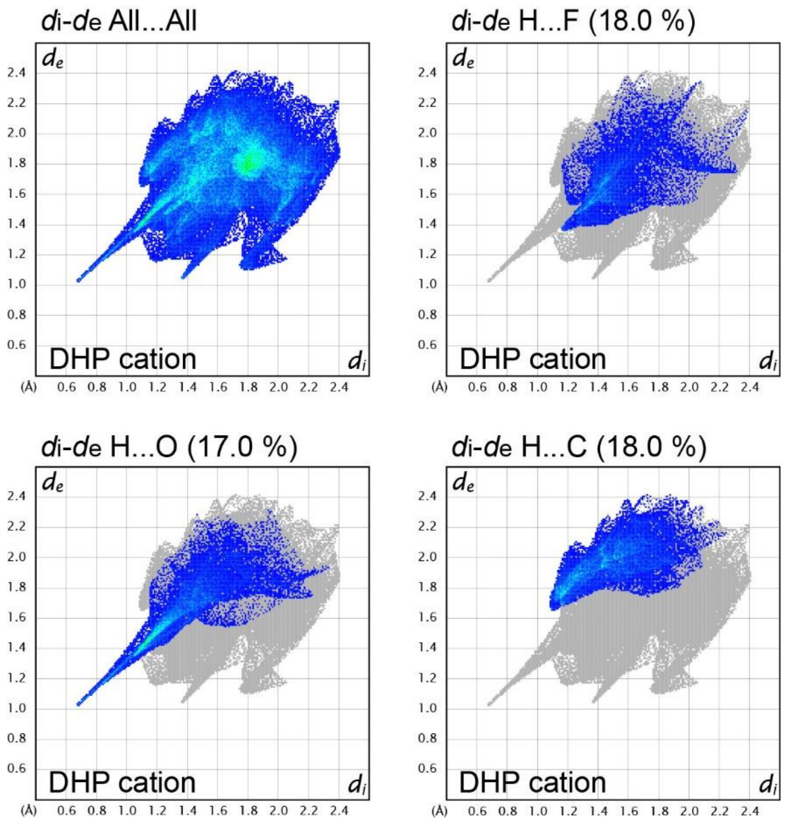

Figure S5. 2D fingerprint plots of the Hirshfeld surface analysis of the DHP cation in DHP-TFS crystals with the atom contacts $d_i \cdots d_e$ of all···all, H···F, H···O, and H···C, corresponding to Figure 2f.

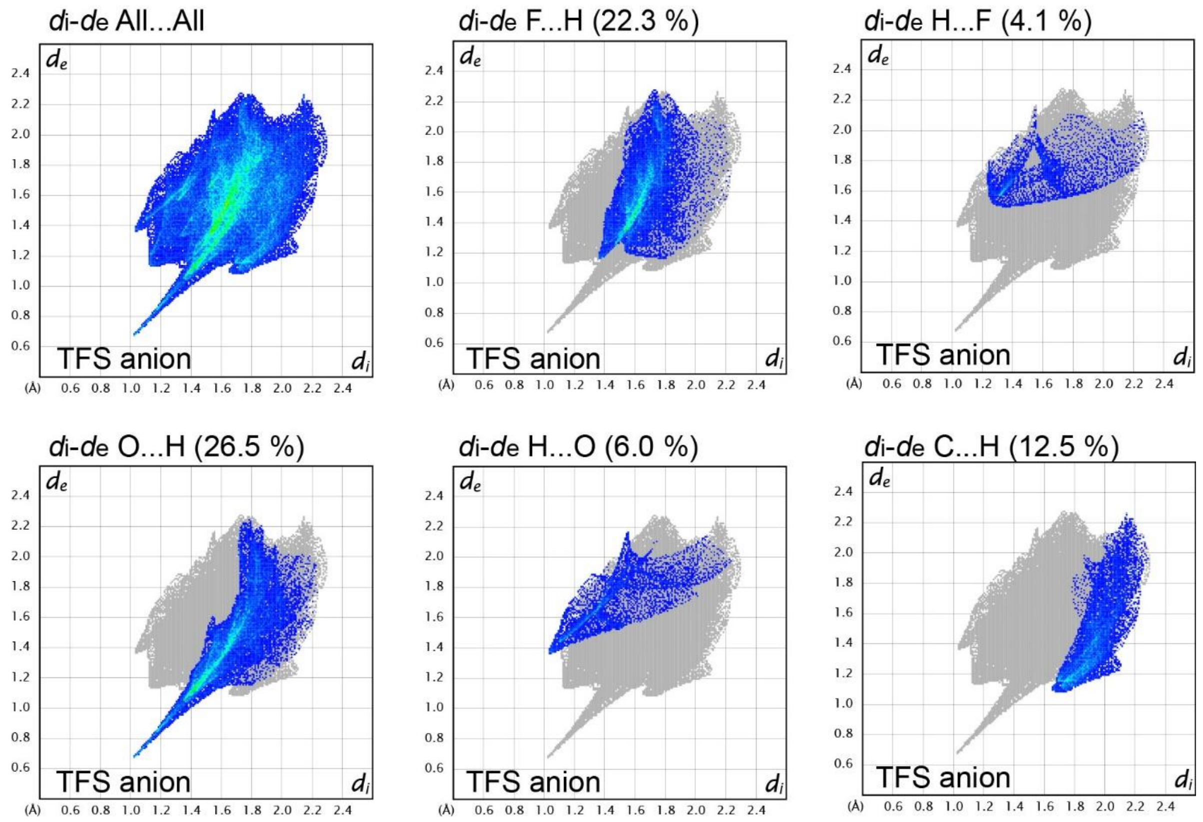

Figure S6. 2D fingerprint plots of the Hirshfeld surface analysis of the TFS anion in DHP-TFS crystals with the atom contacts $d_i \cdots d_e$ of all···all, F···H, H···F, O···H, H···O, and C···H, corresponding to Figure 2f.

E. THz Absorption, THz Refractive Index and THz Wave Generation

For the measurements of the THz absorption and the THz refractive index of the newly developed DHP-TFS crystals, we used the transmission time-domain THz spectroscopy setup as reported in Ref. [*Adv. Photonics Res.* **2021**, 2000098] with a compact femtosecond laser source as a pump laser (38 fs pulse width, central wavelength of 1560 nm, average power of 190 mW, and 100 MHz repetition rate; from Menlo Systems GmbH). THz waves were generated in a 0.70 mm thick DSTMS crystal and detected in a 0.76 mm thick DSTMS crystal (from Rainbow Photonics AG) with well-known characteristics. [*Adv. Photonics Res.* **2021**, 2000098] By analyzing the detected THz signal with and without a 0.15 mm thick DHP-TFS crystal in the THz beam path we were able to determine the refractive index and the absorption coefficient of DHP-TFS in a broadband THz range using the standard extraction procedure by considering a single-pass transmission of the THz pulse in the model for the THz amplitude transmission [*J. Infrared, Millimeter, Terahertz Waves* **2019**, *40*, 395]. The measured results presented in Figure 5 were modeled using the Lorentz multiple oscillator model (see e.g., Equations (1–3) in Ref. [65]) using the same model parameters for both the absorption and the refractive index with the resonances at about 1.46, 2.37, 3.25, 4.71, 5.52, 6.05, 6.75, 7.67, 9.17, 11.01, and 11.65 THz.

For the measurement of the THz-wave generation in the newly developed crystals, we used the above-mentioned setup by employing a 0.15 mm thick as-grown (001) DHP-TFS crystal as a THz generator. The pump beam was incident normally to the as-grown plane and polarized along the polar axis within the crystal plane (along the blue dotted arrow in Figure 2b). For comparison, a standard 1.0 mm (110) ZnTe crystal was also used as a THz generator using the corresponding optimal crystal configuration. [*J. Opt. Soc. Am. B*, **2001**, *18*, 823]. The time-domain signal was acquired with the scanning resolution of 2 fs and the total measured length of the time domain signal was 13.3 ps, resulting in the frequency resolution of 0.085 THz. The time domain signals are shown in Figure 4b and the corresponding Fourier transform spectra in Figure 4a. Figure S7 shows the spectrum detected for DHP-TFS and in addition the corresponding absorption in air and the

absorption coefficients of DHP-TFS generator and DSTMS detection crystal (same as in Figure 2c, up to the measured THz range, e.g., up to about 12 THz for DSTMS). The absorption in % of the air at 296 K, 3 % relative humidity and the interaction length of 30 cm, was calculated from the HITRAN [*J. Quant. Spectrosc. Radiat. Transfer* **2017**, *203*, 3] database, with the resolution of the THz measurement (85 GHz, full line) and with a higher resolution (30 GHz, dotted line).

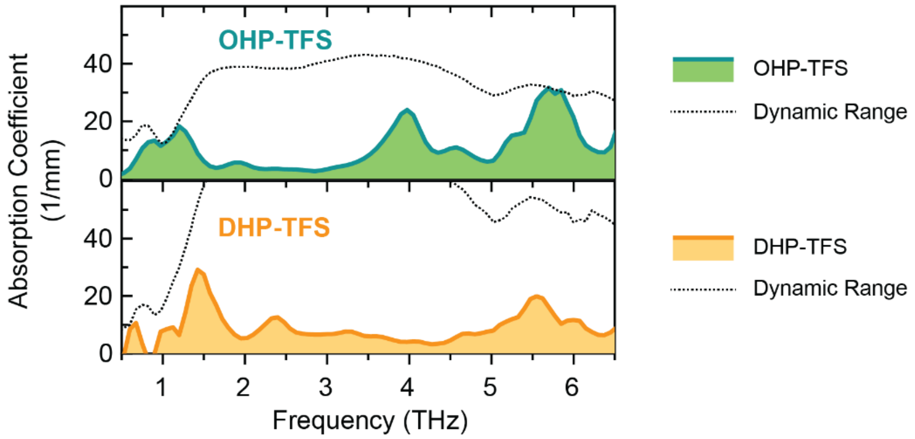

Figure S7. Absorption coefficient of DHP-TFS and OHP-TFS [*Adv. Opt. Mater.*, **2021**, *9*, 2100618] crystals in the THz frequency range. The dotted curve presents the dynamic range for each measurement, i.e., the limit for the measurable absorption coefficient, by considering the corresponding crystal thickness (0.15 mm for DHP-TFS and 0.31 mm for OHP-TFS). Due to the larger thickness of the available OHP-TFS crystals, the amplitude of few peaks for OHP-TFS may be underestimated compared to the measurement of DHP-TFS, while the peaks for DHP-TFS appear below the dynamic range limit.

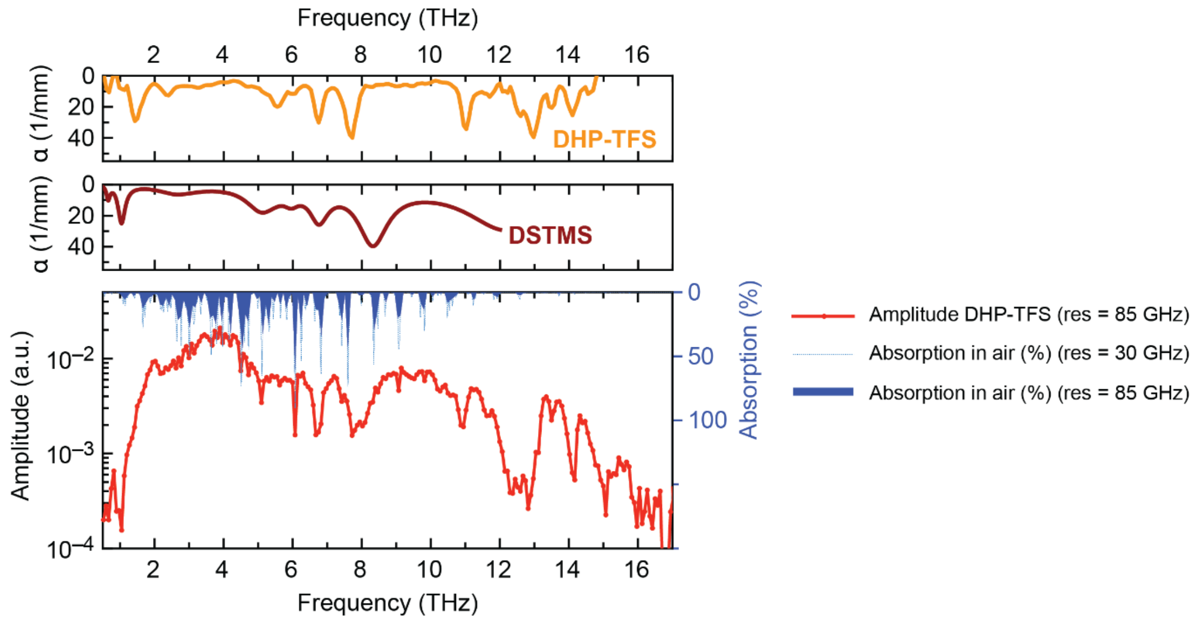

Figure S8. THz wave spectrum as generated in a 0.15 mm thick as-grown DHP-TFS at 1560 nm pump wavelength (red color) and absorption coefficient α of DHP-TFS crystals in the measured 0.5-15 THz range (orange color) and of DSTMS used for detection up to 12 THz (dark red, from literature [*Appl. Sci*. **2019**, *9*, 882]). The THz absorption in air in % at the measurement conditions (relative humidity of 3%) is also shown, as calculated at two different spectral resolutions (30 GHz and 85 GHz).
